# Identifying genes with conserved splicing structure and orthologous isoforms in human, mouse and dog

**DOI:** 10.1186/s12864-022-08429-4

**Published:** 2022-03-18

**Authors:** Nicolas Guillaudeux, Catherine Belleannée, Samuel Blanquart

**Affiliations:** Univ Rennes, Inria, CNRS, IRISA, Rennes, F-35000 France

**Keywords:** Orthology, Transcript orthology, Transcriptome prediction, Alternative splicing, Alternative transcription, Comparative genomics, Spliced CDS, Gene structure

## Abstract

**Background:**

In eukaryote transcriptomes, a significant amount of transcript diversity comes from genes’ capacity to generate different transcripts through alternative splicing. Identifying orthologous alternative transcripts across multiple species is of particular interest for genome annotators. However, there is no formal definition of transcript orthology based on the splicing structure conservation. Likewise there is no public dataset benchmark providing groups of orthologous transcripts sharing a conserved splicing structure.

**Results:**

We introduced a formal definition of splicing structure orthology and we predicted transcript orthologs in human, mouse and dog. Applying a selective strategy, we analyzed 2,167 genes and their 18,109 known transcripts and identified a set of 253 gene orthologs that shared a conserved splicing structure in all three species. We predicted 6,861 transcript CDSs (coding sequence), mainly for dog, an emergent model species. Each predicted transcript was an ortholog of a known transcript: both share the same CDS splicing structure. Evidence for the existence of the predicted CDSs was found in external data.

**Conclusions:**

We generated a dataset of 253 gene triplets, structurally conserved and sharing all their CDSs in human, mouse and dog, which correspond to 879 triplets of spliced CDS orthologs. We have released the dataset both as an SQL database and as tabulated files. The data consists of the 879 CDS orthology groups with their detailed splicing structures, and the predicted CDSs, associated with their experimental evidence. The 6,861 predicted CDSs are provided in GTF files. Our data may contribute to compare highly conserved genes across three species, for comparative transcriptomics at the isoform level, or for benchmarking splice aligners and methods focusing on the identification of splicing orthologs. The data is available at https://data-access.cesgo.org/index.php/s/V97GXxOS66NqTkZ.

**Supplementary Information:**

The online version contains supplementary material available at (10.1186/s12864-022-08429-4).

## Background

Recognising alternative splicing (AS) as the basis of transcriptome and proteome complexity suggests that gene functions should now be investigated at the level of gene isoforms [1]. In this study, we propose a benchmark of highly conserved orthologous genes sharing a common splicing structure and orthologous isoforms sharing common splicing and CDS structures. This high level of conservation suggests a high functional importance and enables to compare gene divergence across species with more details.

AS is a mechanism that produces a variety of transcripts and proteins from a single eukaryotic gene. This mechanism originates from complex regulation processes [1–3] that have been denoted as the “splicing code” [3]. This phenomenon is common in eukaryotic organisms [4]. It is estimated to concern 95% of human multi-exonic genes, with a still growing median of 5 alternatively spliced transcripts per gene [5–8]. Alternative isoforms can show specific interactions with proteins and ligands, specific subcellular locations, tissue-specific expression profiles and differential expression between developmental stages, age and sex [9–14]. Anomalous AS can be associated with both rare and common human diseases [15, 16]. Thus, it is extremely interesting to inventory alternative transcripts at gene level. We actually distinguish two mechanisms leading a gene to produce alternative transcripts. In addition to AS, which consists of splicing introns and yields the mature mRNA, alternative transcription (AT) generates alternative 5’ initiations and/or 3’ terminations during the transcription process.

Orthology is a fundamental concept in computational biology. Orthologous biological characters are considered to have existed in a common ancestor species and are currently shared and derived in its descendants. Orthologs share common inherited phenotypes. While numerous resources are available to identify orthologous genes [17] or exons, very few describe sets of orthologous alternative transcripts. The genome annotation resources and the splice aligners rely on sequence conservation to predict new transcripts sharing homology with already known transcripts. However, this does not correspond to a suitable definition of the transcript orthology, and formal definitions of orthology applying at the alternative transcript level are also scarce. As previously noted by [18], alternative orthologous transcripts are transcribed from orthologous genes and share the same exonic structure: all their exons are orthologous exons. Additionally, alternative orthologous transcripts sharing their coding sequence (CDS) are designated as *spliced CDS orthologs*.

Our study takes us a step further in knowledge concerning splicing orthology. Following on from our earlier work [19], we first provide a formal description of *structural orthology*, applied both at the level of a gene’s splice sites and that of its alternatively spliced transcripts.

Based on this formalism and on highly curated transcripts from CCDS, we then identify a dataset of genes whose splicing structures are conserved across human, mouse and dog. Additionally, a number of spliced CDS orthologs are predicted for the genes through the comparative genomics approach, while known and predicted transcripts of the genes are classified into groups of spliced CDS orthologs that we called CDS orthology groups.

More specifically, we identified a set of 253 orthologous gene triplets in human, mouse and dog, sharing all their splice sites and start and stop codons, and thus identified as structural orthologs (*i.e.* orthologous genes sharing a conserved splicing structure). 879 groups of spliced CDS orthologs were identified for these genes. Orthologous spliced CDSs share the same splicing site structure in each orthologous gene. We gathered evidence for the predicted transcripts using various databases and sequencing datasets. Additionally, we identified a number of transcripts in the dataset as alternative transcripts with distinct UTR regions but having the same CDS, thereby potentially encoding the same protein. Our data are made available for further analysis.

## Results

**Fig. 1:**
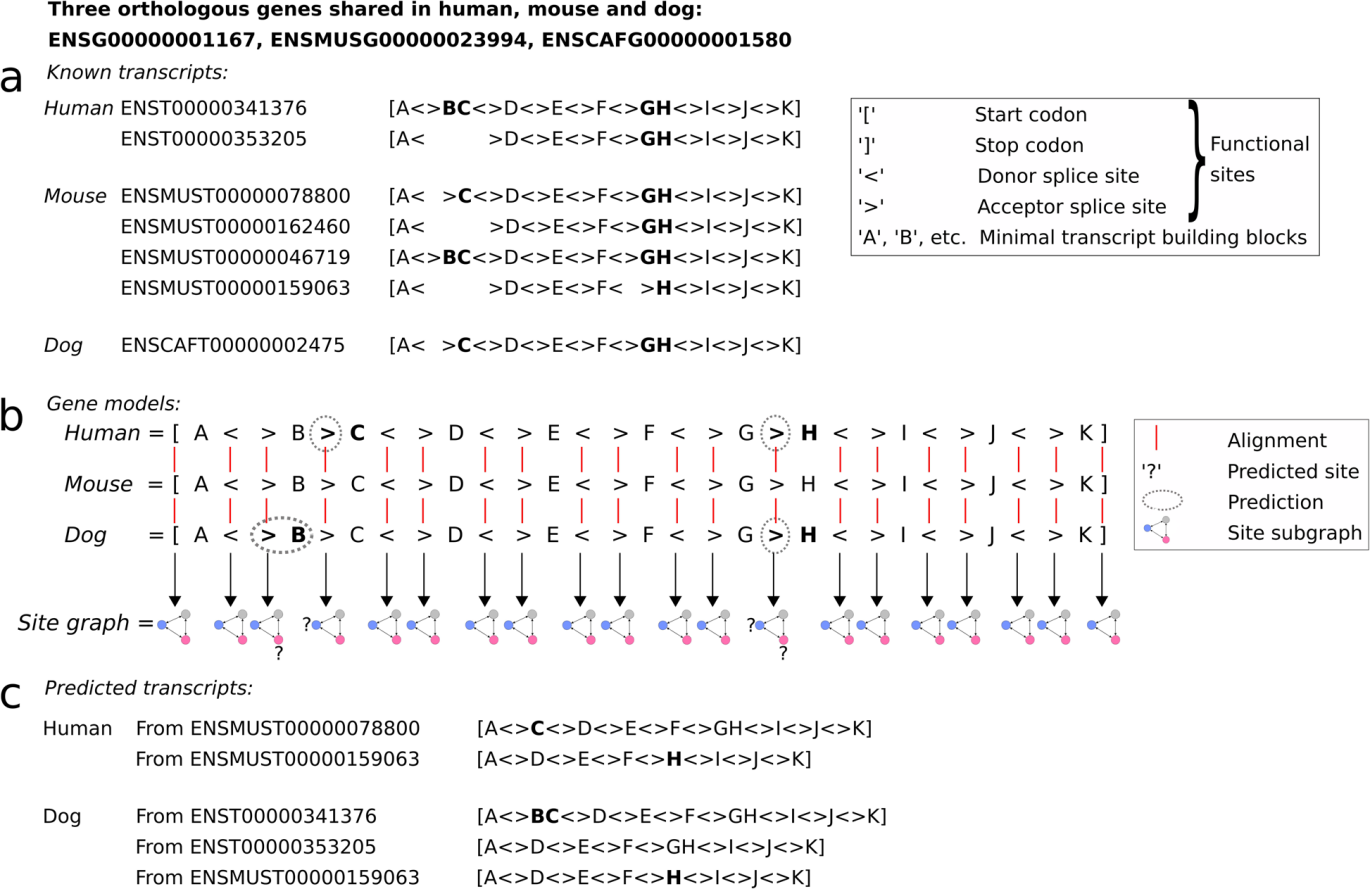
Prediction of new splicing sites and new exons, leading to the prediction of new transcripts. (**a**) Models of known transcripts. They concern the CDS part of the transcripts. They involve *coding blocks*, defining the nucleotidic sequences building a CDS, and *functional sites* delimiting exons on the gene. The coding blocks correspond to the intervals between the functional sites. A same block name occurring in several species indicates a conserved and orthologous region. For example, two transcripts known in mouse involve alternative exons denoted as *C* and *BC*, where both exons contain the block ‘*C*’, and block ‘*B*’ is an alternative 5’ extension of exon *C*. A known human CDS involves an exon *BC* estimated to be an ortholog of the mouse exon *BC*, and the known dog CDS involves an exon *C*, orthologous to the mouse exon *C*. (**b**) Gene model alignment. Each block and site in a gene is aligned with the gene sequence of an orthologous gene, resulting in pairwise gene alignments. These alignments reveal i) the orthology between already known sites (or coding blocks), ii) the sequence homology of known sites (or blocks) with not annotated loci in another gene, resulting in predicted sites and blocks (dashed bubbles). Here, aligning mouse and human genes revealed the presence in human of a homolog of the acceptor site of *C*. This site is thus declared as predicted. It indicates the human gene is able to express the *C* exon alone, without the ‘*B*’ part. Additional predictions: acceptor site of *H* in human and dog, and ‘*B*’ block plus its acceptor site in dog. The *site graph* summarizes pairwise orthology relations: a node is a functional site and an edge is an orthology relationship. (**c**) Predicted transcripts. Five transcripts are made possible from site and block predictions

In this study, we developed a comparative genomics method based on a description of coding exon structures across multiple species. The method first identified splicing sites conserved among orthologous genes, thereby denoted as *orthologous splicing sites*. Next, the orthologous genes were compared according to the orthologous splicing sites, in order to estimate whether each splicing site involved in a known transcript has an ortholog in another species. If so, a transcript sharing a conserved splicing structure was identified in the other species, and it was denoted as an *orthologous transcript*. Finally, we identified orthologous genes sharing a conserved splicing structure: all their splicing sites are conserved over the considered species. These genes were denoted as *structurally orthologous genes* (see “Methods” and Fig. 1 as an example). More precisely, in addition to splicing sites, start and stop codons were also considered, collectively defined in the paper as *functional sites*. The coding sequences (CDS) specifically were compared, and thus *spliced CDS orthologs* were predicted.

The study focused on 2,167 genes shared in human, mouse and dog and their transcripts, which were stringently chosen. These genes were selected so as to exhibit several complete alternative transcripts, each having a manually curated annotation in human and mouse according to the CCDS database. Among them, we identified 253 triplets of structurally orthologous genes, which share all their functional sites and have all their CDSs conserved across the three species. 879 triplets of spliced CDS orthologs were identified among these genes: the 879 distinct CDSs expressed in a given species have orthologs in the two others, thus none of the CDS is specific to a species, nor missing in any of the three species.

### Transcript prediction : 6,861 predicted CDSs

**Fig. 2:**
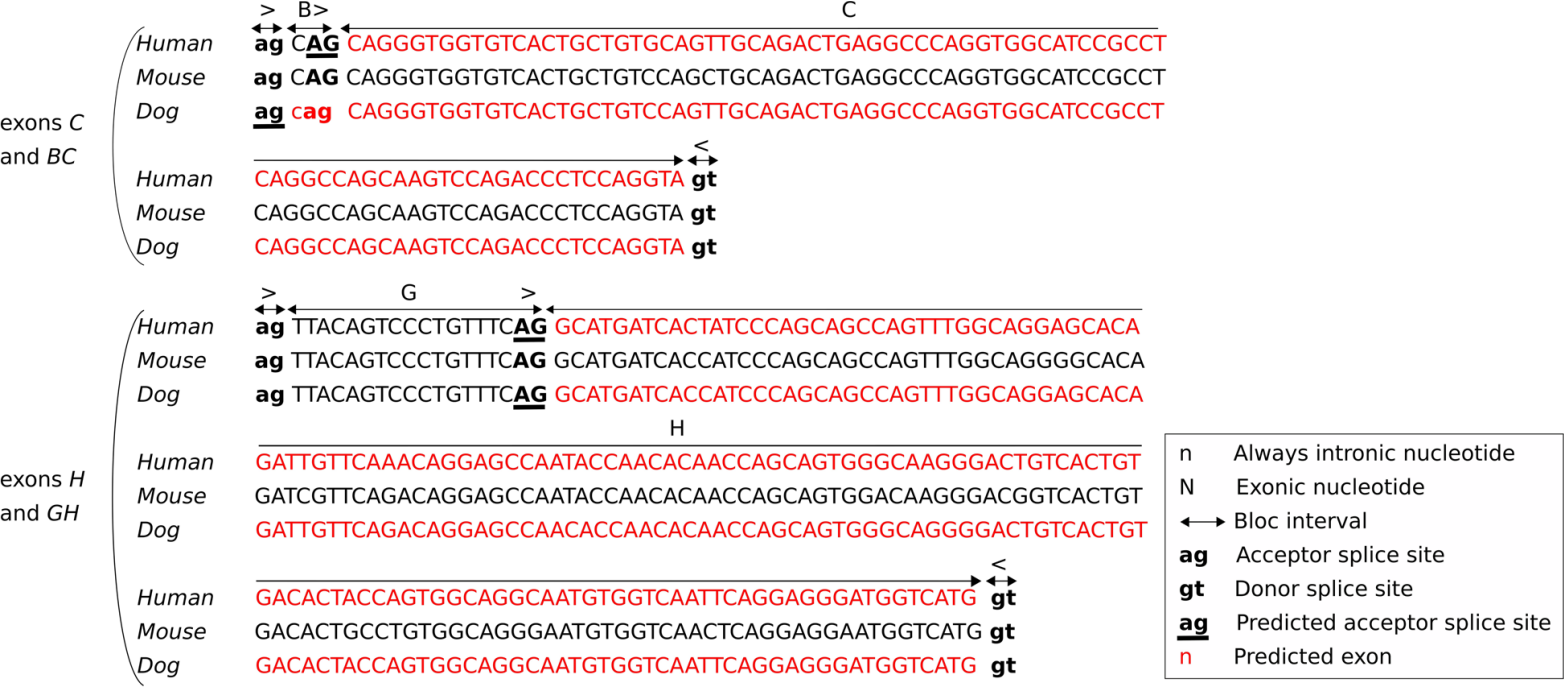
Details of a sequence alignment: prediction of acceptor sites and blocks on human and dog. Two multiple alignments are shown here, providing more details about the predictions illustrated in Fig. 1: the alignments of the loci encoding exons *C* and *BC*, and exons *H* and *GH*. Uppercases indicate nucleotides involved in known exons, and lowercases indicate intronic nucleotides never observed yet to belong to an exon. For example, in human and mouse genes, the longer exon *BC* is known (uppercase). In the dog gene however, only the shorter exon *C* is known and the upstream nucleotides have been observed as intronic so far (lowercase). Note that the exon *C* is not known in human (it does not occur in any human transcript, see Fig. 1a). The splicing sites are indicated in bold, and predicted splicing sites are underlined. For example, a motif “*AG*” in human has been aligned with the known acceptor “*AG*” of exon *C* in mouse, thereby yielding a predicted orthologous splicing site in human (underlined bold “*AG*”). This motif is now identified as an acceptor site of exon *C* in human. Additionally, a motif “*ag*” in dog has been aligned with the known “*ag*” acceptor of exon *BC* in mouse, yielding a predicted orthologous splicing site in dog (underlined bold “*ag*”). The nucleotides of the predicted exons are shown in red. For example, a motif “*cag*” in dog has been aligned with the sequence “*CAG*” of the known block ‘*B*’ in mouse, yielding a predicted block (red “*cag*”). As a result, shorter exons *C* and *H* can exist in human (only longer exons *BC* and *GH* were known). In dog two new exons can exist, *H* (only *GH* was known) and *BC* (only *C* was known)

The 2,167 orthologous genes shared in human, mouse and dog express 18,109 known transcripts. Models of their spliced CDSs were built, making possible comparisons of alternative CDSs across species (see an example in Fig. 1a). The pairwise gene comparisons led, on the one hand, to predict orthology relationships between the functional sites involved in the known transcripts of both species, and on the other hand to predict new candidate functional sites and exons. In the example shown Fig. 1b, several exons observed in mouse CDSs had no orthologs known in the human and dog CDSs, but the corresponding orthologous splicing sites and exons could be predicted in the human and dog genes. Both latter predictions relied on pairwise sequence alignments of each exon and splicing site in one gene with the complete sequence of another gene. Figure 2 illustrates such sequence alignments and the prediction of conserved splicing sites.

Based on the known and predicted exons, and on the transcript structure comparisons, we identified orthology relationships between functional sites and between CDSs. In the example illustrated in Fig. 1, the four predicted exons in human and dog led to predict orthologs of the four CDSs that are known in mouse. Orthologous CDSs have identical transcript models (see Fig. 1c and “Methods”). This way, we predicted 6,861 CDSs in human, mouse and dog (Table 1), each being the ortholog of a known transcript CDS. Thus, the predicted number of transcripts represented 38% of the known transcripts initially considered. In a later section, we provide additional evidences for some of the predictions.

**Table 1:** Amount of CDSs predicted by comparative genomics

Species	*Human*	*Mouse*	*Dog*
Known transcripts in ENS90data	8,374	6,511	3,224
Predicted CDSs from human	-	1,878	2,251
Predicted CDSs from mouse	1,223	-	958
Predicted CDSs from dog	317	234	-
Total of predicted CDSs	1,540	2,112	3,209
Total number of transcripts	9,914	8,623	6,433

### Prediction distribution across model species and emergent model species

Predictions are not equally distributed across species, reflecting differences in the initial amount of knowledge considered. Because human is the most widely documented species (8,374 known transcripts considered), it garners the lowest number of CDS predictions (1,540 predicted CDSs, Table 1). Thus, although there is less room to complement a highly studied transcriptome, it would be possible to improve its current annotations by better accounting for alternative transcripts identified in less studied transcriptomes. As expected, dog is the least documented species (3,224 known transcripts) and it receives the largest number of predicted CDSs (3,209). This is congruent with the general task of comparative genomic approaches, consisting in transferring transcript annotations from well documented model species to the less documented non-model or emergent model species.

### A set of 253 structurally orthologous genes in human, mouse and dog

A functional site graph links its orthologous functional sites (splice sites and start and stop codons) for each gene triplet. These graphs allow us to compare gene structures across the three species (see “Methods” and Fig. 1b as an example).

From 2,167 orthologous gene triplets, 1,661 were retained for subsequent analysis. The genes considered comprised exclusively of either functional sites specific to one species, or of functional sites shared in two or three species (see “Methods”).

Among the 1,661 gene triplets, 253 yielded functional site graphs displaying all the functional sites shared in all three species, and were defined as *structurally orthologous genes* (Fig. 1b). The other genes displayed at least one functional site specific to a species, or shared in two out of three species.

The following hypothesis can be made concerning each structurally orthologous gene identified: when all splice sites and coding exons are conserved, all three orthologous genes should be able to express the same CDSs, and then the same proteins. Conversely, no CDS should be specific to, or missing in any species.

### Transcript orthology : 253 genes with all the orthologous CDSs conserved across human, mouse and dog

#### genes with all orthologous cDSs shared in a single copy for each species

**Fig. 3:**
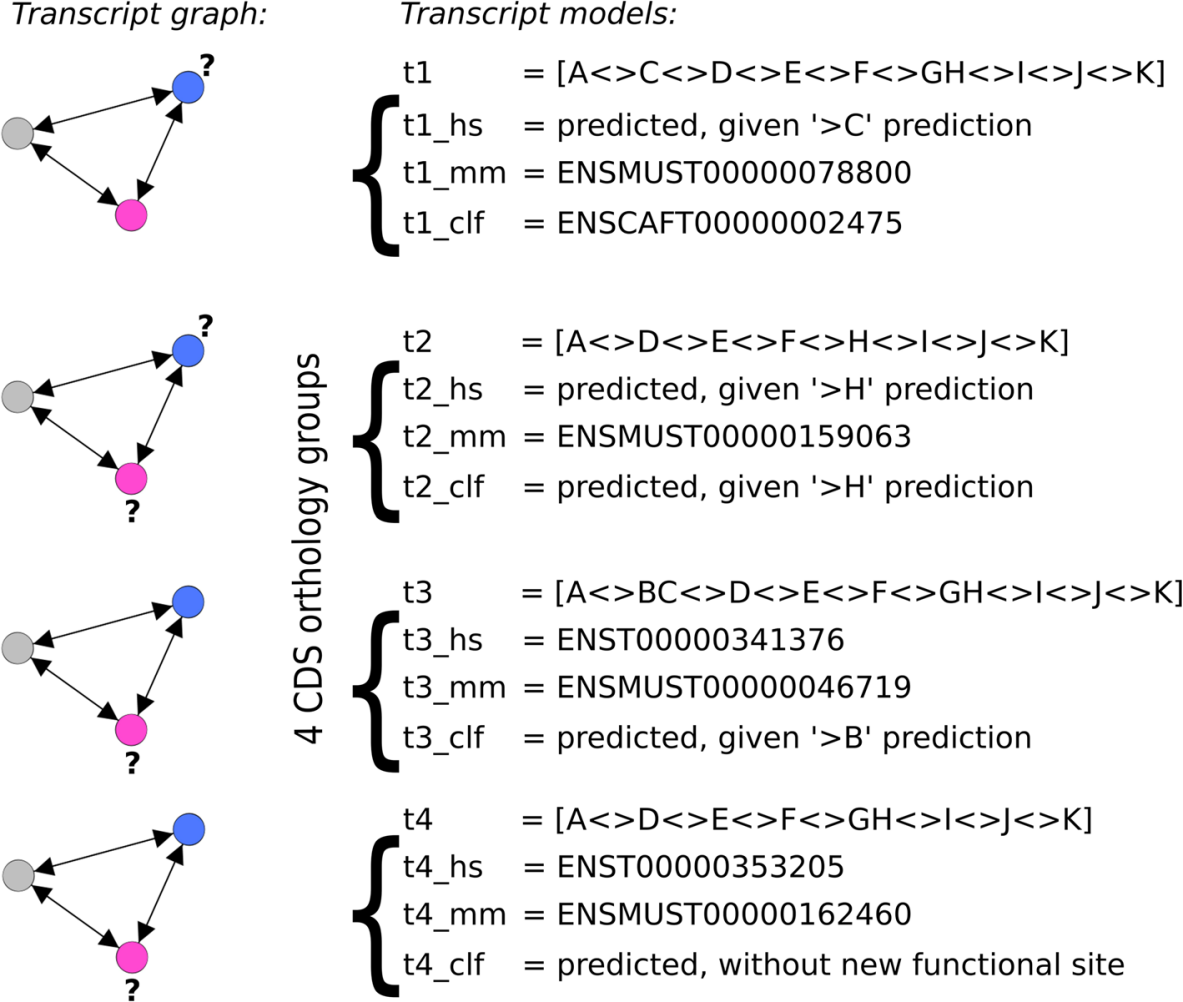
Identification of CDS orthology groups: case of a gene whose transcripts are conserved over species. This figure concerns the gene presented in Figs. 1 and 2. Comparing its gene models over human, mouse and doc (see Fig. 1b) serves as a basis i) to compare the structures of CDSs across species (see Fig. 1a), ii) to predict orthologous CDSs using predicted exons and sites (see Fig. 1c), and iii) to establish orthology relations between CDSs (left of Fig. 3). Thus, the resulting *graph of transcripts* connects transcripts sharing the same CDS splicing structure, thereby identifying CDS orthology groups. Left of Fig. 3, the transcript graph for the orthologous genes *ENSG*00000001167,*ENSMUSG*00000023994 and *ENSCAFG*00000001580 contains 4 subgraphs, each being a triplet of orthologous CDSs (nodes are CDSs, blue for human, grey for mouse and red for dog, with “ ?” indicating a predicted CDS). This implies that i) all the seven known gene transcripts, in human, mouse and dog actually represent four different CDS splicing structures, ii) five new CDSs are predicted to make the graph complete, iii) for this gene, each of the four CDS splicing structures is feasible in human, mouse ang dog, so the three genes share the same orthologous CDSs. Right of Fig. 3, the four braces indicate details of the four CDS orthology groups, showing the Ensembl identifiers of known transcripts, the predicted CDSs, and the spliced CDS structures t1 t2 t3 and t4 shared over the three species

Using the 1,661 gene triplets retained for gene structure analysis, a transcript graph per gene triplet (see “Methods”) was built in order to draw orthology links between CDSs and to define groups of orthologous CDSs (denoted as *CDS orthology groups*, Fig. 3).

We first considered gene triplets with a transcript graph exclusively containing CDSs shared in a single copy for each species (see “Methods”). 986 genes fulfilled this requirement. Among them, 135 genes had all their CDSs shared in all three species in a single copy per species. Following the classical “one to one” definition of orthology, each species displayed the same spliced CDS structure in a single copy, so the ancestor probably already possessed this spliced CDS structure. The 135 genes in question expressed a total of 462 CDS orthology groups (triplets of spliced CDS orthologs), involving 845 known and 541 predicted CDSs in human, mouse and dog genes (Table 2, set S135). An example of such a gene triplet displaying exclusively orthologous CDSs is shown in Fig. 3.

**Table 2:** Encoded CDSs in the subset of 253 structurally orthologous genes

Dataset	Transcripts	Human	Mouse	Dog	Total
S253	Known transcripts	854	762	280	1,896
	Predicted CDSs	180	249	600	1,029
S135	Known transcripts	364	331	150	845
	Predicted CDSs	98	131	312	541
S118	Known transcript	490	431	130	1,051
	Predicted CDSs	82	118	288	488
	Redundant CDSs	155	132	1	288
	Missing UTRs	25	10	66	101
S114	Set with redundancy	109	103	1	213

As expected, all 135 gene triplets belong to the set of 253 structurally orthologous genes, with conservation across all three species of all the required functional sites and of all the coding exons, allowing each species to form the same orthologous spliced CDS structures (see “Methods”).

#### genes with CDSs in multiple copies for at least one species: 213 CDSs with variable UTR

**Fig. 4:**
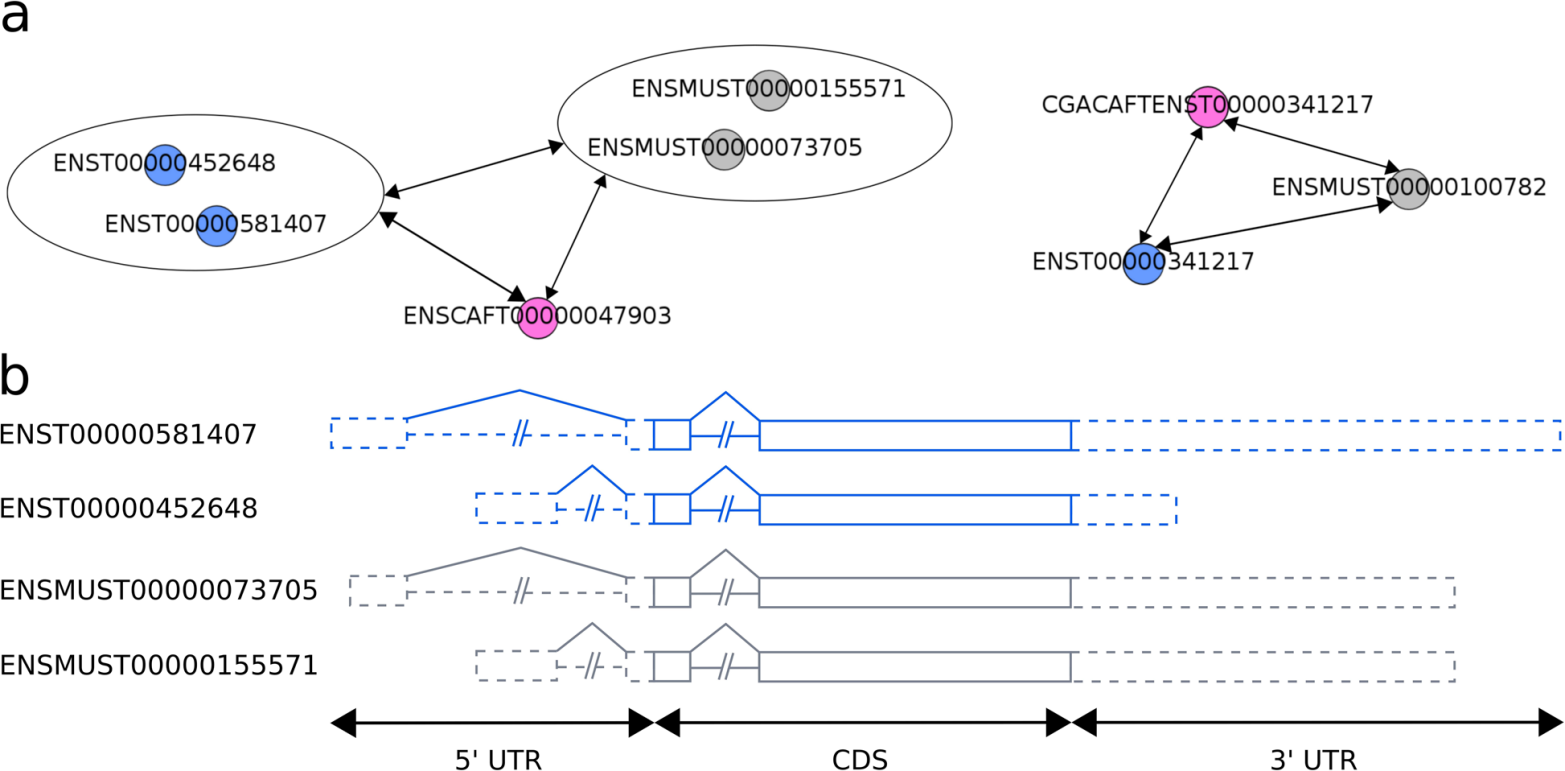
Transcript graph of a gene showing several alternative transcripts encoding a same CDS. The Ensembl identifiers of orthologous genes are *ENSG*00000173065,*ENSMUSG*00000037750 and *ENSCAFG*00000031142. These are identified as orthologous genes structurally conserved, sharing a common gene model: [*A*<>*B*>*C*[*D*]. (**a**) The transcript graph obtained comprises two sugraphs: one is a triplet (right of Fig. 4a), and the other is made up of more than three nodes (left of Fig. 4a). In fact, *ENST*00000452648 and *ENST*00000581407 transcripts in human (blue nodes) encode the same CDS with a structure model [*ABCD*]. Both transcripts have different UTR regions resulting from alternative transcription initiation and termination, as illustrated in Fig. 4b. This is also the case for the *ENSMUST*00000155571 and *ENSMUST*00000073705 transcripts in mouse (grey nodes). (**b**) Representation of the UTR exons from the alternative transcripts encoding the same CDS. Coding exons are shown as boxes and UTR exons as dashed boxes. Introns are shown as lines. The bottom of the figure shows the gene regions, including the 5’UTR, CDS and 3’UTR exons

Among the 253 structurally orthologous genes however, 118 (253-135) did not conform to the previous properties. Each of the 118 transcript graphs was such that it contained at least one CDS being redundant in a species: two or more known transcripts in this species encoded the same CDS (see Additional file 5). An example of such a gene is shown in Fig. 4, where human and mouse each have two known transcripts with an identical CDS, but distinct UTR regions.

The 118 genes expressed a total of 1,051 known transcripts which led us to infer 488 predicted CDSs. Known transcripts displayed 288 redundant CDSs (Table 2, set S118) and 417 distinct CDS orthology groups could be observed overall. Whenever CDSs were redundant in a species, a one-to-one relation of orthology between transcripts (CDS plus UTR) did not apply. For example, in Fig. 4, CDS redundancy exists at the transcript level in human and mouse, and we cannot determine which of the two human transcripts is orthologous to which of the two mouse transcripts based on the CDS alone. However, shared CDSs are unique and the orthology definition still applies at CDS level, i.e. to the genes’ protein isoforms. Each of the spliced CDS structures encode an orthologous protein *a priori* shared in the three species, and the 118 genes’ ancestors presumably expressed ancestors of these 417 protein isoforms.

The redundant CDS cases mainly correspond to alternative transcripts with a same CDS but different 3’ or 5’ UTR regions. Among the 1,051 known transcripts of the 118 gene sets, 101 were found to lack 5’ or 3’ UTR, or both (Table 2). We do not take them into consideration in the following enumeration of CDS redundancy. We found 114 gene triplets from 118 displaying redundant CDSs in at least one species such that the underlying transcripts are all described together with their UTR regions. We assume that such transcripts are genuine cases of multiple alternative transcripts encoding a same CDS. This represents a total of 213 sets of known transcripts encoding redundant CDSs. While 109 and 103 sets are respectively identified in human and mouse, only 1 is identified in dog. This disparity most likely results from the lack of information in the emergent model species rather than from the absence of CDS redundancy (Table 2, set S114).

### Experimental evidence for predicted transcripts

In this section, our previous predictions are substantiated with additional sources. Please note that the precision and recall measures of the applied comparative genomics method can be found in [19]. We examined seven additional databases in order to validate our transcript predictions using knowledge not included in the present study (see “Methods”). We detail here the results concerning the 253 structurally orthologous genes in human, mouse and dog. The additional databases are the Ensembl 96, Ensembl 98, Ensembl 102, Ensembl 103, UCSC, XBSeq and FEELnc databases. An important number of our predictions were substantiated, representing up to 42.8% of validated predictions in dog and around 20% of validated predictions in human and mouse (Table 3). Overall, 350 predicted CDSs (34%) were validated (*i.e.* tagged as *confirmed*) from the additional databases.

**Table 3:** 253 gene triplets: predicted CDSs found in databases

Species	*Human*	*Mouse*	*Dog*
Number of predicted transcripts	180	249	600
Found in Ensembl 96 data	27	43	0
Found in Ensembl 98 data	+2	+1	+183
Found in Ensembl 102 data	+0	+1	+0
Found in Ensembl 103 data	+3	-	+0
Found in XBSeq data	+8	+5	-
Found in UCSC data	+2	+1	+0
Found in FEELnc data	-	-	+74
Total of found CDSs	42	51	257
	23.3%	20.5%	42.8%

For the 679 remaining predicted transcripts that were not found in additional databases, we sought evidence in RNA-seq sequencing datasets by looking for signatures of predicted transcripts in the sequence reads. We considered an exon junction specific to this transcript as a predicted CDS signature, in other words, a junction that is not observed in any known transcript from the input data (set ENS90data, see “Methods”). 394 of the 679 predicted transcripts (58%) contained at least one specific exon junction (see Table 4), while all their specific junctions were identified in the reads for 255 of them (64.7%). These transcripts were tagged as *achievable*.

**Table 4:** 253 gene triplets: predicted CDSs with specific exon junctions found in read data

Species	*Human*	*Mouse*	*Dog*
Predicted CDSs not found in databases	138	198	343
Without specific exon junctions	59	78	148
With specific exon junctions	79	120	195
With aligned reads	47	61	147
CDSs found with reads	59.5%	50.8%	75.4%

Finally, we managed to find hints of 89 (49.4%) predicted transcripts in human, 112 (45%) in mouse and 404 (67.3%) in dog (see Tables 3 and 4, see also Additional file 6). Thus, we found evidence for the existence of 58.8% of our transcript predictions derived from our comparative genomics method, suggesting that the method is suitable for CDS prediction. The type of confirmation obtained for each predicted transcript was kept in the database as an attribute (see Methods and Availability).

### Description of the 253 structurally orthologous genes

The 253 triplets of genes we defined as structurally orthologous have, by definition, all their start/stop codons and splice sites conserved over human, mouse and dog. According to the Gene Ontology, most of these genes belonged to the categories “cellular process”, “biological regulation” and “metabolic process” (see Additional file 8). These genes express 1,896 known transcripts and we predicted 1,029 additional CDSs (Table 2, set S253, and see Additional files 1–3.). An average of 2.5 (1,896/(3 ×253)) known transcripts per gene was expressed, ranging from 1 (in dog) to 13 (in human). We predicted an average of 1.3 (1,029/(3 ×253)) CDSs per gene, ranging from 1 (in each species) to 12 predictions (in dog). Among the 1,029 predicted CDSs, 350 were found in other databases and 255 had specific exon junctions that were aligned with sequencing reads.

Each of the 253 orthologous genes encoded the same spliced CDS structures in the three orthologs. Gene proteome is shared across species and we identified 879 (462 in the S135 set, and 417 in the S118 set) CDS orthology groups. The gene transcriptomes may differ, however, due to multiple transcripts encoding the same CDS, with a potentially different number of such alternative transcripts across species. We identified 114 genes from the 253 where such CDS redundancy occured (45% of genes), which involved 213 sets of redundant CDSs. According to our data, alternative transcripts encoding a same CDS represent a tangible situation as 8% (213/(3 ×879)) of the sets of CDSs contain at least two different transcripts with distinct UTR regions. The phenomenon could be higher than 8% with regard to the genes in our dataset. In particular UTR regions in dog are almost undocumented at present, leading to just 1 observation among known transcripts (Table 2, set S114).

## Discussion

We applied a comparative genomics approach to a set of 2,167 genes in order to compare CDSs and gene structures between the three species: human, mouse and dog. We predicted CDSs in all three species and found that about 15% (253/1,661) have orthologous splicing structures that are wholly conserved in human, mouse and dog, and so could express the same set of isoforms over the three species. These *structurally orthologous* genes are defined as having conserved all start/stop codons and splice sites (denoted as the *functional sites*). For these genes, we found additional annotated and experimental data supporting 59% of the predicted CDSs, underpinning the robustness of our results. These data could be useful in several kinds of analyses.

### Alternative transcripts encoding a same CDS

A recent study showed that alternatively spliced transcript diversity and expression levels across human tissues are mostly driven by AT start and stop sites [20]. Here we document such cases of AT where several alternative transcripts encode a same protein. Multiple transcripts encoding the same CDS occur in 45% of the structurally conserved genes, concerning 8% of the CDSs. This suggests a widespread phenomenon. It may be assumed that various alternative promoters and different 3’UTR regions yield as many different possibilities to regulate a given protein expression, depending on the required specificity of the tissues physiology. Interestingly, some studies have reported that a given protein isoform may or may not be expressed, depending on the transcribed UTR regions [21]. These observations and our results thus suggest that, even if the same functional sites are shared between orthologous genes, genes may express different transcriptomes with different UTR regions or different numbers of transcripts encoding a same CDS. These redundant alternative transcripts may be involved in responses to different regulatory processes.

### Benchmark for a spliced aligner

Sequence homology lies at the heart of numerous protein and transcript predictions. However, there is still room for improvement in the underlying comparative genomics and spliced alignment methods [22]. The latter work shows recurrent challenges in accurately identifying intron-exon boundaries, and in handling non canonical *GT* and *AG* splice sites. The latest spliced aligner algorithms consider the spliced structure of a query transcript and the known splice sites of the target gene, thereby searching explicitly for spliced orthologs [23]. Our sets of spliced orthologs can be used to test such methods using real data.

### Comparative genomics of regulatory elements

Our study formally defines orthology at splice site level, providing a more in-depth examination of the conservation of gene sequences located within intronic and exonic regions, and implied in the alternative splicing regulation. Indeed, if all splice sites are conserved and their orthology identified, it becomes possible to interpret sequence divergence in the surrounding regions that could be involved in the regulation of alternatively spliced transcript expression. Recent studies have shown that AS events follow conserved patterns of expression shared across species [24, 25], indicating an underlying conserved mechanisms and regulatory sequences related to the genes’ splicing programs. Additional observations show that some splicing events encounter divergence in their inclusion rates [26] or divergence in their tissue specific expression rates [27], which alternatively suggests regulatory sequences divergence.

### Comparative transcriptomic at alternatively spliced transcript level

Finally, our description of orthology at spliced CDS level may be useful in comparative transcriptomic studies, helping to identify the differential expression of orthologous alternative CDSs across human, mouse and dog species. However, most current studies in comparative transcriptomics focus either on the gene level, taking into consideration all the alternatively spliced transcripts expressed collectively, or at exons’ junction level, ignoring both the complete AS combinations forming an alternatively spliced transcript and all the different alternatively spliced transcripts, possibly involving a given AS event [28]. We believe that formal identification of orthologous alternatively spliced transcripts is thus lacking in current comparative transcriptomic studies.

## Conclusion

In this paper, we apply a comparative genomics method based on the identification of the coding exon and splicing site structure of genes and the identification of the spliced CDS structure of transcripts. We define orthology at the functional site level of genes, identifying orthologous start and stop codons, donor and acceptor splice sites, and then at CDS level, identifying CDS orthology groups. These formalisms help to both predict new CDSs, and to identify genes sharing a same structure and transcriptome across species.

Applying a selective approach with the objective of producing highly reliable data, we studied a set of 2,167 orthologous genes shared in human, mouse and dog from CCDS and Ensembl. Given these genes, we predicted 6,861 CDSs, almost doubling the knowledge available in an emergent model species, the dog. We identified a set of 253 orthologous genes sharing all their functional sites and all their CDSs across the three species. We called the latter genes *structural orthologs*. We predicted 1,029 CDSs for the 253 genes, confirming 59% of them using additional annotation and experimental data. From these genes, we identified 879 CDS orthology groups (see Additional file 4). Interestingly, among the 2,637 gene CDSs, 8% were encoded by two or more alternative transcripts with different UTR regions. This concerned 45% of the genes examined, suggesting an important role for alternative transcription in the data considered.

Our data consists of 879 groups of spliced CDS orthologs which are available in the form of a SQL database as well as tabulated files. They are useful for research focusing on the identification of splicing orthologs and research focusing on genes conservation and divergence across species. This covers comparative transcriptomics at the level of orthologous alternatively spliced transcripts, for instance, and comparative genomics at the level of splicing regulation sequences.

## Methods

### Data sources: gene triplets on human, mouse, dog

The selected genes were one-to-one orthologous genes shared by human, mouse and dog species as defined in Ensembl release 90 [29], based on GRCh38.p10, GRCm38.p5 and CanFam3.1 assembly versions. Additionnaly, to be selected, a gene triplet must have at least two alternatively spliced transcripts in CCDS [30] for human and mouse, and at least one Ensembl transcript for dog. Thus, all human and mouse transcripts are obtained from CCDS, and all dog transcripts are obtained from Ensembl. Such sequences are called *known transcripts*. The resulting set contained a total of 2,167 orthologous gene triplets, expressing 18,109 known transcripts (8,374 in human, 6,511 in mouse and 3,224 in dog). This dataset is referred to in the rest of the paper as “ENS90data”.

### A database of orthologous genes and transcripts structurally conserved in human, mouse and dog

An SQL database, Transcript_ortho, displays the gene and transcript structures of 253 orthologous gene triplets conserved in human, mouse and dog species (see Results). The genes proposed in Transcript_ortho are structurally orthologous in the three species. The transcript_ortho tables describe the intron/exon composition of the genes and transcripts, through their genomic positions, as well as the orthology relationships for both coding exons and CDSs. Transcript_ortho contains 2,925 transcripts, along with their known (1,896) or predicted (1,029) status. In the second case, an additional attribute indicates the degree of confidence in the prediction, through an experimental confirmation tag (see below in the “Assessing Evidence” paragraph). The database, its schematic diagram (see Additional file 7) and the complete set of predicted transcripts, in GTF format, can be downloaded at https://data-access.cesgo.org/index.php/s/V97GXxOS66NqTkZ.

### Representation of genes and transcript structures: structure model definitions

In Eukaryotes, a precursor transcript is composed of exons and introns. Each intron is delineated by two splice sites, the splice donor site (generally “*GT*”) and the splice acceptor site (generally “*AG*”), which allows intron excision during the splicing process. The exon sequences, remaining after splicing, constitute the mature transcript, or messenger RNA (mRNA). The mRNA contains a coding DNA sequence (CDS) to be translated into a protein. A *CDS* is composed of a succession of codons (trinucleotides), starting with a start codon (generally “*ATG*”), ending with a stop codon (“*TAG*”, “*TGA*” or “*TAA*”), and including no in-frame stop codon in between.

We used the formalism described in [31] and [19] to represent this structure, allowing us to model the structure of a gene from the intron/exon structure of its known transcripts.

**Fig. 5:**
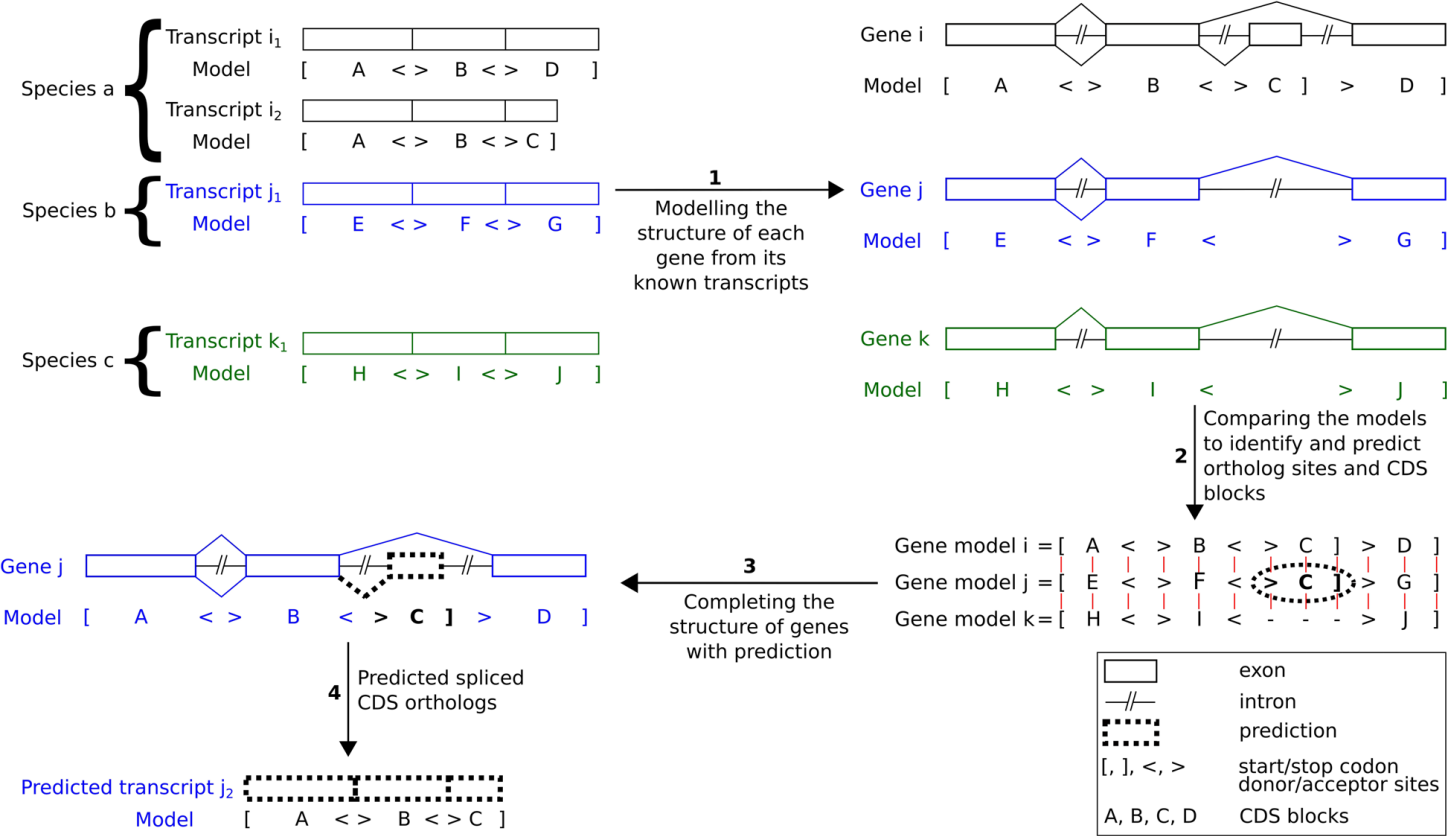
Identification of the structural conservation of a gene and prediction of CDSs. The method takes known transcripts as input, and then outputs gene and transcript structure models, predicted CDSs and structural orthology relations between CDSs. Top left: the spliced structures of five transcripts known in three species. (1) The known transcripts of a gene allow us to build its intron/exon structure. (2) Pairwise alignments of each functional site and coding block of a gene against another gene sequence allows us i) to identify the orthology between known sites and blocks between two species (orthologous elements are represented in a same column with red lines) and ii) to reveal previously unknown functional sites and coding blocks (dashed box). (3) In each gene, the orthologous coding blocks receive a common name. Renamed blocks of gene *j* are shown. (4) Revealed elements serve as a basis to predict orthologous CDSs. Here, gene *j* gained the prediction of the CDS *j*_2_, which is a spliced ortholog of CDS *i*_2_ known in gene *i*. Indeed, as the ‘*C*’ coding block and flanking sites (‘ >*C*]’) were revealed in gene *j*, the *i*_2_ transcript appears feasible in gene *j*. No CDS ortholog of the *i*_2_ transcript is predicted in gene *k*, since the ‘*C*’ coding element was not found in gene *k*, making the *i*_2_ transcript unfeasible in gene *k*

The *gene structure model*$M^{G}_{i}$ of a gene *i* is composed of a list of *N* tokens *K*_*i*,*m*_, each corresponding to either one of the above mentioned functional sites or to a protein coding block (an exonic block). The *functional sites* are the donor (represented as: ‘ <’) and acceptor (‘ >’) splice sites, and the start (‘ [’) and stop (‘ ]’) codons. The *coding blocks* (represented by alphabetical characters, ‘*A*’, ‘*B*’, etc.) correspond to the protein coding exons observed in the known and predicted (see below) transcripts. For instance, the structure model of a given gene *i* can be represented as $M^{G}_{i} = [A < > B < > C] > D]$ (see Fig. 5).

The *transcript structure model*$M^{T}_{i,u}$ of a transcript *T*_*i*,*u*_ expressed in gene *i* represents the structure of the CDS part of the transcript (e.g. $M^{T}_{i,1} = [A<>B<>C]$ and $M^{T}_{i,2} = [A<>B<>D]$). $M^{T}_{i,u}$ is composed of a subset of tokens *K*_*i*,*m*_ from $M^{G}_{i}$, where first and last tokens are start and stop codons and the other tokens (the alphabetical letters) stand for the coding blocks representing the exonic segments that constitute the CDS, each exon being delineated with its splicing sites. Technically, each token is associated with its genome coordinates, and the gene structure model is obtained from the structure models of its known transcripts [19]. For instance, the $M^{G}_{i}$ gene model above could have been obtained from the two transcript models $M^{T}_{i,1}$ and $M^{T}_{i,2}$ (see Fig. 5).

### Pairwise comparison between orthologous genes: structural orthology definitions

#### Comparing two gene structure models: functional site prediction

Given two orthologous genes *i* and *j* in two species, together with their known transcripts, each gene structure model $M^{G}_{i}$ and $M^{G}_{j}$ is firstly drawn from the structure of its known transcripts. The pairwise comparison between $M^{G}_{i}$ and $M^{G}_{j}$ thus consists of examining whether each token *K*_*i*,*m*_ from $M^{G}_{i}$ (and vice-versa for the tokens *K*_*j*,*n*_ from $M^{G}_{j}$) is conserved or not in the orthologous gene *j*, which is done by aligning coding blocks of gene *i* against the genomic sequence of gene *j* (see [19] for more details). If the sequence of a token *K*_*i*,*m*_ is aligned in gene *j*, either it corresponds to an already known token *K*_*j*,*n*_ in $M^{G}_{j}$, or it does not. In the latter case, a token is added to complete $M^{G}_{j}$, referred to as a *predicted orthologous token* (*cf.* the ‘ >’, ‘*C*’ and ‘ ]’ tokens in Fig. 5, predicted from gene *i* to gene *j*). In cases where tokens *K*_*i*,*m*_ and *K*_*j*,*n*_ represent orthologous coding blocks, they are unified by a same letter.

It is worth mentioning that, given the dataset considered, a site or an exon predicted in gene *j* belongs to none of the known transcripts of that gene. In fact, a site/exon predicted in gene *j* from gene *i* corresponds to a conserved sequence shared by the nucleotidic sequence of the two genes, associated with a known site/exon belonging to at least one of the known transcripts of gene *i*. This highlighting of new exons through a comparative genomics approach paves the way for transcript prediction (Fig. 5). The pairwise gene comparison leads to the following definitions of structural orthology.

Two aligned tokens, *K*_*i*,*m*_ from gene *i* and *K*_*j*,*n*_ from gene *j*, define a pair of *orthologous tokens*, denoted as $\mathcal {A}(K_{i,m}, K_{j,n})$ (and reciprocally $\mathcal {A}(K_{j,n}, K_{i,m})$, the alignment relation of the two tokens being symmetrical). In the case of coding blocks, they are denoted by a same letter.

Two genes *i* and *j* whose structure models $M^{G}_{i}$ and $M^{G}_{j}$ contain only pairs of orthologous tokens $\mathcal {A}(K_{i,m}, K_{j,n})$ define a pair of *structurally orthologous genes*. $M^{G}_{i}$ and $M^{G}_{j}$ are syntactically equal.

Two transcripts *T*_*i*,*u*_ from gene *i* and *T*_*j*,*v*_ from gene *j* whose structure models $M^{T}_{i,u}$ and $M^{T}_{j,v}$ contain only pairs of orthologous tokens $\mathcal {A}\left (K_{i,m}, K_{j,n}\right)$ define a pair of structurally orthologous transcripts, named *spliced CDS orthologs*. $M^{T}_{i,u}$ and $M^{T}_{j,v}$ are syntactically equal.

#### Comparing two transcript structure models: transcript prediction

The comparative approach based on structure models allows us to compare the transcriptomes of two orthologous genes in order to determine the structural orthology relation between transcripts and to predict transcripts. A pairwise comparison between transcripts of two orthologous genes assessed whether each transcript *T*_*i*,*u*_ from gene *i* (and reciprocally for transcripts *T*_*j*,*v*_ from gene *j*) has a spliced CDS ortholog in the orthologous gene *j*, in other words, whether a transcript *T*_*j*,*v*_ exists in *j* with the same CDS structure as $T_{i,u} (\text {in other words},\ M^{T}_{i,u}=M^{T}_{j,v})$.

If so, we infer a CDS orthology relationship between the known transcripts *T*_*i*,*u*_ and *T*_*j*,*v*_. Otherwise, it is possible to examine whether each token involved in transcript model $M^{T}_{i,u}$ has an orthologous token in the gene *j* model $M^{G}_{j}$. If so, we predict that a new transcript for gene *j* is possible, with the same spliced CDS as transcript *T*_*i*,*u*_. Below are the formal definitions:

Given two orthologous genes *i* and *j* and a transcript *T*_*i*,*u*_ from gene *i*, if each token of $M^{T}_{i,u}$ has an orthologous token in the gene model $M^{G}_{j}$, then a sequence homologous to *T*_*i*,*u*_, referred to as $\mathcal {S}\left (M^{T}_{i,u}, M^{G}_{j}\right)$, is assessed to be expressible in *j*. The $\mathcal {S}\left (M^{T}_{i,u}, M^{G}_{j}\right)$ sequence consists of the concatenation of sequences in gene *j* designated as structurally orthologous of the coding blocks involved in $M^{T}_{i,u}$. In Fig. 5, for example, the transcript labelled “2” in gene *i* is formed of exons *A*, *B* and *C*. All these exons have orthologs in gene *j*, where exons *A* and *B* are known, and exon *C* is predicted. Thus, the $\mathcal {S}\left (M^{T}_{i,2}, M^{G}_{j}\right)$ sequence is composed of a concatenation of the gene *j* sequences denoted as *A*, *B* and *C*.

If, additionally, the resulting sequence $\mathcal {S}\left (M^{T}_{i,u}, M^{G}_{j}\right)$ forms a CDS then the transcript model $M^{T}_{i,u}$ is called *executable* in *j* given the gene model $M^{G}_{j}$, which is denoted by $\mathcal {E}\left (M^{T}_{i,u}, M^{G}_{j}\right)$. To form a CDS, the start and stop codon in the $\mathcal {S}\left (M^{T}_{i,u}, M^{G}_{j}\right)$ sequence of *T*_*j*,*v*_ are separated by a number of nucleotides being a multiple of three, and the sequence do not contain any inframe stop codon. This indicates that a transcript *T*_*j*,*v*_, with the CDS sequence $\mathcal {S}\left (M^{T}_{i,u}, M^{G}_{j}\right)$ and the CDS structure $M^{T}_{i,u}$ (i.e. *T*_*j*,*v*_ and *T*_*i*,*u*_ are spliced CDS orthologs), is expressible in gene *j*. For example, in Fig. 5, transcripts labelled “2” in genes *i* and *j* are considered as spliced CDS orthologs since they share the same model $M^{T}_{i,2}=M^{T}_{j,2}=[A<>B<>C]$. Moreover, the concatenated sequences *A*, *B* and *C* from *j* form a CDS in gene *j*. Thus, $M^{T}_{i,2}$ is *executable* in gene model $M^{G}_{j}$: $\mathcal {E}\left (M^{T}_{i,2}, M^{G}_{j}\right)$. Roughly speaking, the transcript *T*_*i*,2_ can also exist in *j*.

If this executable transcript, denoted as *T*_*j*,*v*_, does not already belong to the set of known gene *j* transcripts under consideration, then it is called a *predicted transcript*. For example, in Fig. 5, the transcript labelled “2” in gene *j* is a predicted transcript, involving the predicted exon *C*.

By the end of the pairwise comparison between two orthologous genes *i* and *j*, we thus dispose of a pairwise alignment of gene models $M^{G}_{i}$ and $M^{G}_{j}$, orthology relationships between tokens, $\mathcal {A}(K_{i,m}, K_{j,n})$, a set of predicted transcripts, and orthology relationships between transcripts (i.e., $M^{T}_{i,u}= M^{T}_{j,v}, \mathcal {E}\left (M^{T}_{i,u}, M^{G}_{j}\right)$ and $\mathcal {E}\left (M^{T}_{j,v}, M^{G}_{i}\right)$), with transcripts *T*_*i*,*u*_ and *T*_*j*,*v*_ being known or predicted. In the paper, the term *CDS*, or *spliced CDS*, refers to the protein coding transcript region (excluding the UTR), underscoring the fact that our predictions are based only on the coding parts of the transcripts.

### Comparison across multiple orthologous genes

#### Comparing gene structures across three species: graph of functional sites

As described above, a pairwise comparison between two orthologous genes *i* and *j* produces pairs of orthologous tokens, $\mathcal {A}(K_{i,m}, K_{j,n})$, and tokens with no identified ortholog, denoted as $\mathcal {A}(K_{i,m}, -)$, where “ −” stands for a gap. For each token, this defines whether it is shared by both genes, or is specific to one gene, respectively. Thus, given three orthologous genes *i*, *j* and *k* in three species, a token shared by the three species can be identified via three pairwise orthologies: $\mathcal {A}(K_{i,m}, K_{j,n}), \mathcal {A}(K_{i,m}, J_{k,o})$, and $\mathcal {A}(K_{j,n}, K_{k,o})$, indicating that {*K*_*i*,*m*_,*K*_*j*,*n*_,*K*_*k*,*o*_} is a triplet of orthologous tokens. In order to represent such a three-species comparison between structural elements at gene triplet level, we defined a *graph of functional sites*, $\mathcal {G}^{FS}$.

**Fig. 6:**
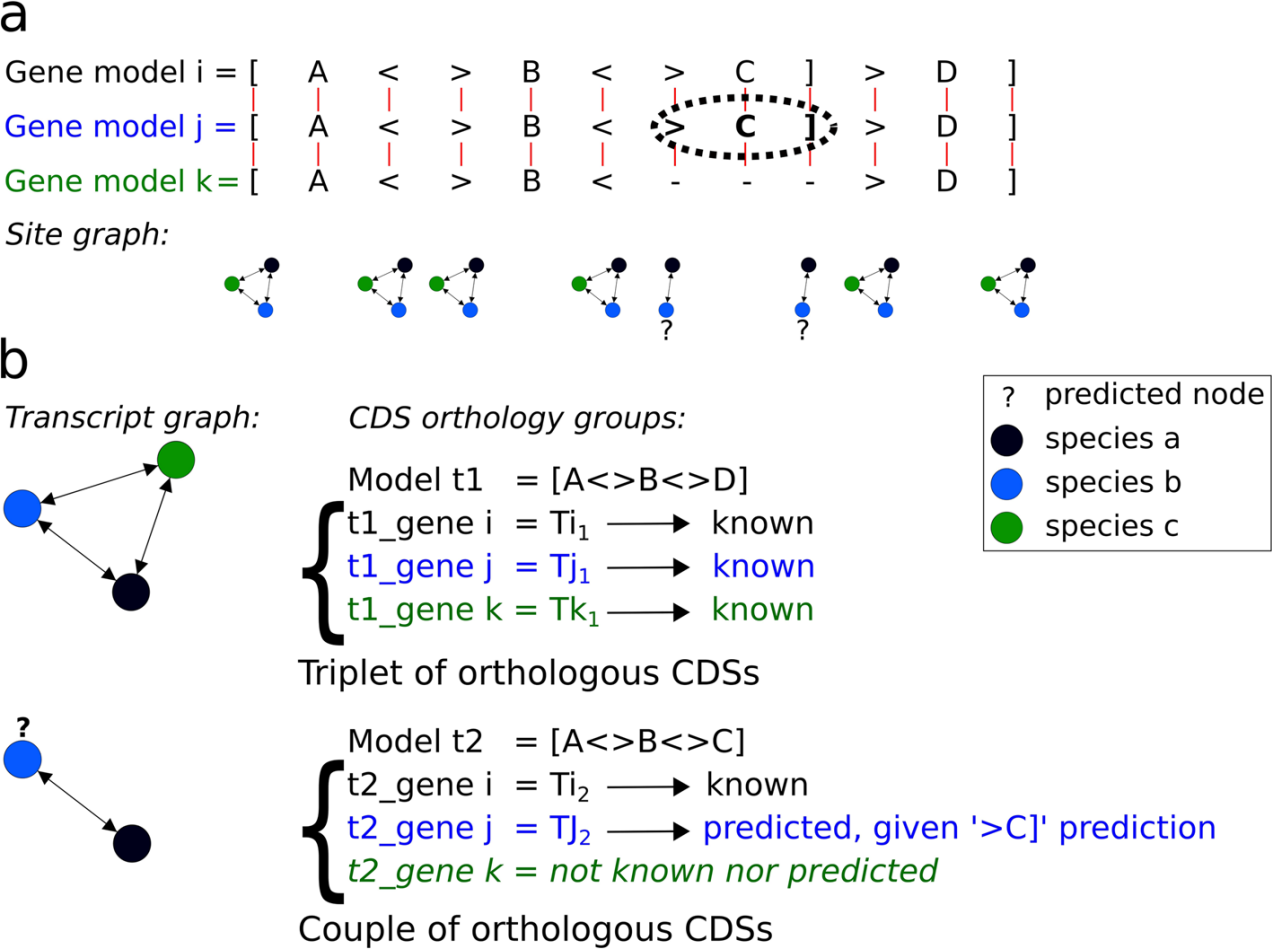
Site graph and transcript graph: identification of CDS orthology groups across three species. (**a**) The pairwise alignment of gene models indicates site and block orthology relationships and predicts new feasible elements (*e.g.*, block ‘*C*’ and its flanking sites ‘ >*C*]’ in gene *j*, see Fig. 5). The orthology relations are linked in a multi-species site graph (bottom of Fig. 6a), where nodes are functional sites, edges are orthology relations, and ‘ ?’ indicates predicted sites. (**b**) Determination of CDS orthology groups. Transcript graphs were built using pairwise transcript orthology. The example illustrates two CDS orthology groups. A first group involves three known orthologous CDSs. A second group of two CDSs is made up of a known CDS in gene *i* and a predicted CDS in gene *j* (‘ ?’ symbol). Gene *k* cannot form the third orthologous CDS due to the lack of an element in the gene

Each node of $\mathcal {G}^{FS}$ is labelled with a token corresponding to a functional site of one of the three genes. All the functional sites involved in known and predicted transcripts are taken into consideration to build the graph. Each edge of $\mathcal {G}^{FS}$ connects a token *K*_*i*,*m*_ of a gene *i* to a token *K*_*j*,*n*_ of another gene *j* iff $\mathcal {A}(K_{i,m}, K_{j,n})$ (Fig. 6a). We built a graph of functional sites per triplet of orthologous genes in three species.

#### Comparing transcript structures across three species: graph of transcripts, to reveal CDS orthology groups

A similar structure was designed to compare gene transcripts across three species, the *graph of transcripts*, $\mathcal {G}^{T}$. We built a graph of transcripts per triplet of orthologous genes in three species. Each node of $\mathcal {G}^{T}$ corresponds to a transcript (either known or predicted) in one of the three species. Each edge of $\mathcal {G}^{T}$ connects a transcript *T*_*i*,*u*_ of a gene *i* to a transcript *T*_*j*,*v*_ of a gene *j* iff they are spliced CDS orthologs, *i.e.*, $M^{T}_{i,u}=M^{T}_{j,v}$ (Fig. 6b).

#### Graph analysis: identifying structurally orthologous genes and conserved transcriptomes in three species

Three types of subgraph are considered in a functional site graph $\mathcal {G}^{FS}$ : a singleton corresponds to a functional site present in only one species, a couple represents a site shared by two species, while a triplet represents a site shared by all three species (Fig. 6a). Only gene triplets where functional site graphs are made up of singleton, couple and triplet subgraphs, are considered for subsequent analysis. A transcript graph $\mathcal {G}^{T}$ is thus obtained for these genes.

A functional site graph containing only triplets of sites indicates that each functional site has an orthologous site in each of the other species. Such a gene has a structure shared in all three species, which suggests that the structure was already existing in their common ancestor, defining a triplet of *structurally orthologous genes*. A transcript graph $\mathcal {G}^{T}$ containing only triplets of transcripts implies that each CDS has an orthologous CDS in each of the other species. This indicates that each of the three orthologous genes can express the same CDS set.

### Assessing evidence for predicted transcripts from annotations and experimental data

From the annotated transcripts contained in our ENS90data base set, a number of predicted transcripts are generated by our comparative genomics method. We assessed how these predictions are supported by complementary transcript annotations and experimental data. By the end of the process, each predicted transcript had been tagged with one of four labels: *confirmed*, *possible*, *achievable* or *not achievable*.

#### Predicted transcripts found in annotation databases

Each predicted transcript *T* was first sought from additional annotations issued from four databases: UCSC (version June 2019, human, mouse and dog, [32]), Ensembl release 96, release 98, release 102 (human, mouse and dog) and release 103 (human, dog), XBSeq (human and mouse, [33]) and FEELnc (dog, [34]).

If one of the databases contained a spliced CDS, described in GTF format and corresponding to the coding exons of *T*, then *T* was tagged as *confirmed*.

#### Identifying exon junctions specific to predicted transcripts in read data

Unconfirmed predicted transcripts were examined against RNA-seq raw data. We considered comprehensive datasets spanning a large quantity of tissue in human [35], mouse [25] and dog [34], and searched for hints of a predicted transcript, defined as *specific exon junctions*, among the reads. A given exon junction was defined as specific to a given predicted transcript if no other occurrence of that junction belonged to the transcripts considered in our initial ENS90data set. Finding reads which contain the specific exon junctions of a transcript does not prove that the complete transcript was expressed in the sequenced data, but it nonetheless highlights the presence of a signature of the predicted transcript.

Three potential results were considered. i) If the transcript contained no specific exon junction, the predicted transcript was tagged as *possible*. ii) If specific exon junctions were identified in the transcript but not all were covered by aligned reads, the predicted transcript was tagged as *not achievable* according to the read data considered. iii) If specific exon junctions were identified and they were all covered by aligned reads, the predicted transcript was tagged as *achievable* according to the read datasets.

## Supplementary Information

**Additional file 1** Full gene information in human with known and predicted transcripts for the 253 gene set. The additional file is an excel spreadsheet consisting of a sheet which describes all the information of a standard GTF file.

**Additional file 2** Full gene information in mouse with known and predicted transcripts for the 253 gene set. The additional file is an excel spreadsheet consisting of a sheet which describes all the information of a standard GTF file.

**Additional file 3** Full gene information in dog with known and predicted transcripts for the 253 gene set. The additional file is an excel spreadsheet consisting of a sheet which describes all the information of a standard GTF file.

**Additional file 4** List of the 879 orthologous CDS groups with their transcript identifiers. The additional file is an excel spreadsheet consisting of a sheet with, for each orthologous CDS group, its transcript identifiers in the three species.

**Additional file 5** Size of each CDS orthology group concerning the 118 gene set (alternative UTRs). The additional file is an excel spreadsheet consisting of a sheet with, for each orthologous gene triplet and each CDS orthology group, the number of transcripts encoding a same CDS per species.

**Additional file 6** List of the 1,029 predicted transcripts and their associated evidence found in external databases. The additional file is an excel spreadsheet consisting of a sheet with, for each predicted transcript, its evidence tag (see “Methods”).

**Additional file 7** Relational diagram of the transcript_Ortho SQL database. The additional file is at the PDF format.

**Additional file 8** Figure of the gene ontology matches for the 253 human genes. The additional file is at the PDF format.

## Data Availability

The data analyzed come from the CCDS [30] and Ensembl 90 databases [29] (December 2018). The data used to verify our predictions come from the UCSC [32] (June 2019), Ensembl [29] (release 96, 98, 102, and 103), XBSeq [33], and FEELnc [34] databases for the annotation verification, and from published read data sets [25], [34], and [35] for the experimental verification. Our resulting data are freely available at https://data-access.cesgo.org/index.php/s/V97GXxOS66NqTkZ. The repository displays i) the CDS orthology groups for each out of the 253 gene triplets over human, mouse and dog, ii) the predicted transcripts, associated with their experimental evidence score, and iii) the SQL Transcript_ortho database.

## Abbreviations

AS: Alternative splicing
AT: Alternative transcription
CCDS: Consensus coding sequence
CDS: Coding sequence
DNA: Deoxyribonucleic acid
FEELnc: Flexible extraction of lncRNAs
GTF: Gene transfer format
mRNA: messenger RNA
RNA: Ribonucleic acid
RNA-seq: RNA sequencing
UCSC: University of California Santa Cruz
UTR: Untranslated region
