# Supplementary figures and images for: Identifying genes with conserved splicing structure and orthologous isoforms in human, mouse and dog
Source: BMC Genomics. 2022 Mar 18;23:216. doi: 10.1186/s12864-022-08429-4 (PMC8933948; doi:10.1186/s12864-022-08429-4)

# Additional file 7 --- Relational diagram of the Transcript\_Ortho SQL database.

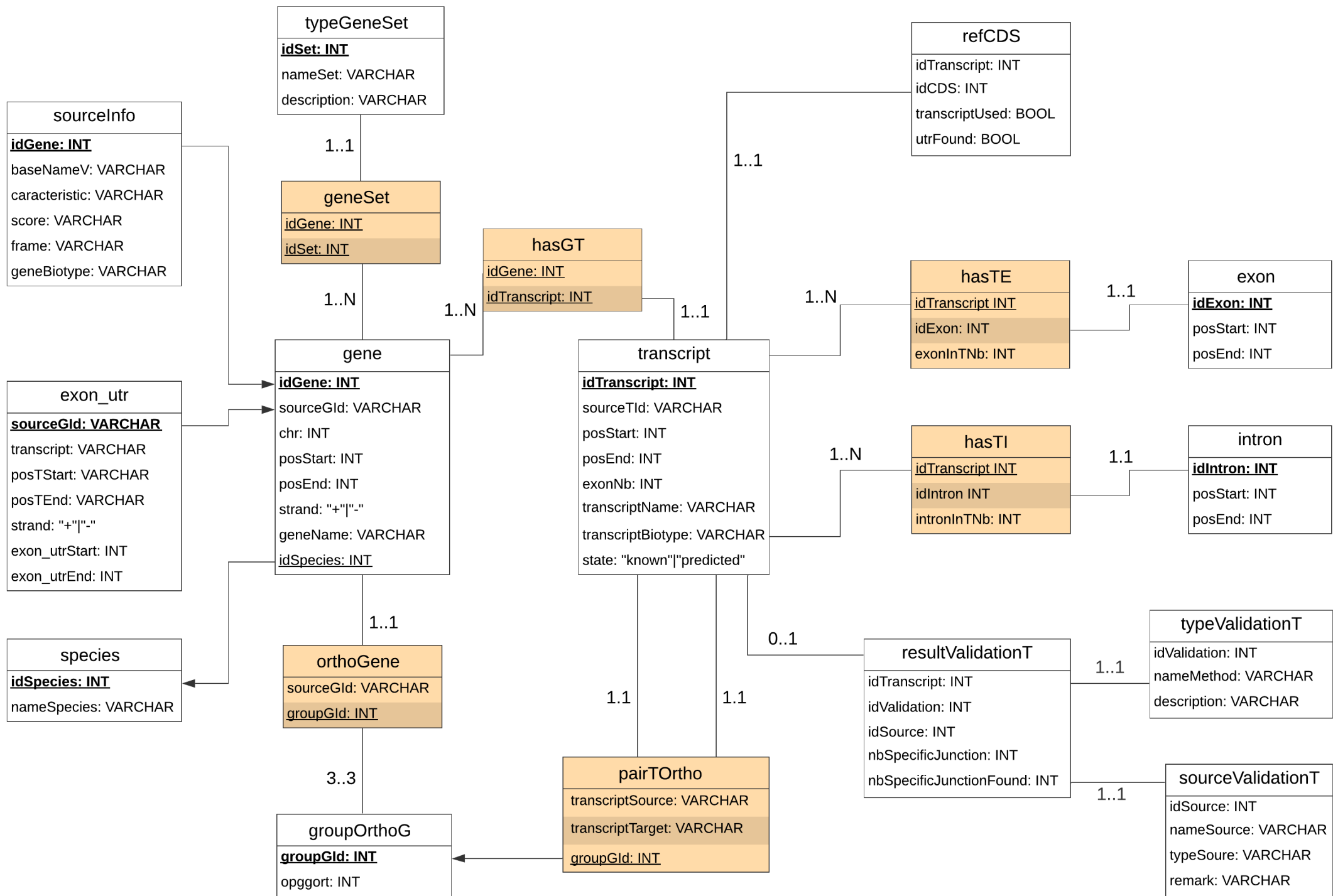

Supplement: Supplementary file 7 — Additional file 7 Relational diagram of the transcript_Ortho SQL database. The additional file is at the PDF format. [file 12864_2022_8429_MOESM7_ESM.pdf]
