# Supplementary material for: Identifying genes with conserved splicing structure and orthologous isoforms in human, mouse and dog
Source: BMC Genomics. 2022 Mar 18;23:216. doi: 10.1186/s12864-022-08429-4 (PMC8933948; doi:10.1186/s12864-022-08429-4)

Additional file 8 --- Figure of the Gene Ontology matches for the 253 human genes.

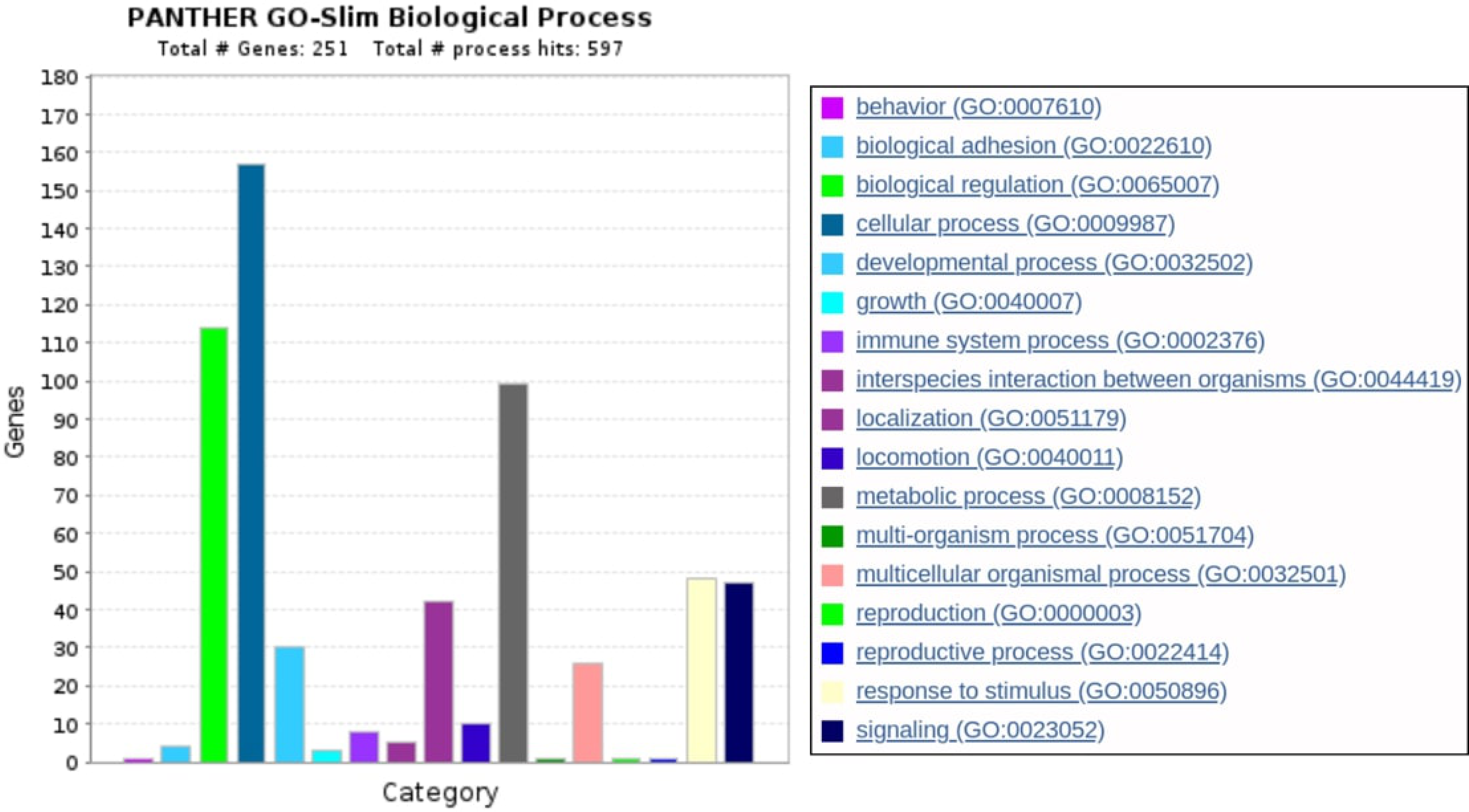

Supplement: Supplementary file 8 — Additional file 8 Figure of the gene ontology matches for the 253 human genes. The additional file is at the PDF format. [file 12864_2022_8429_MOESM8_ESM.pdf]
